# Importancia de la técnica de perfusión intracardiaca para los estudios *in vivo* en odontología

**DOI:** 10.21142/2523-2754-1101-2023-148

**Published:** 2023-03-26

**Authors:** Mauricio Andre Zapata-Sifuentes, Kiyoko Suzuki-Barrera, Angela Quispe-Salcedo

**Affiliations:** 1 Division of Anatomy and Cell Biology of the Hard Tissue, Department of Tissue Regeneration and Reconstruction, Niigata University Graduate School of Medical and Dental Sciences. Niigata, Japan. mzapata@dent.niigata-u.ac.jp, kiyoko.suzuki.barrera@gmail.com, aquispesa@dent.niigata-u.ac.jp Division of Anatomy and Cell Biology of the Hard Tissue Department of Tissue Regeneration and Reconstruction Niigata University Graduate School of Medical and Dental Sciences Niigata Japan mzapata@dent.niigata-u.ac.jp kiyoko.suzuki.barrera@gmail.com aquispesa@dent.niigata-u.ac.jp

**Keywords:** estudios in vivo, perfusión intracardiaca, histología, *in vivo* studies, intracardiac perfusion fixation, histology

## Abstract

Los estudios *in vivo* en odontología exigen un alto nivel de precisión, ya que implican la experimentación con un ser viviente y un comprensivo análisis histológico para validar la hipótesis planteada. Sin embargo, el proceso mediante el cual se obtienen las láminas histológicas que serán estudiadas suele ser, muchas veces, erróneamente minimizado. Para obtener cortes histológicos de alta calidad y que estos sean capaces de reaccionar favorablemente a técnicas inmunológicas más complejas, es necesario preservar o “fijar” los tejidos de interés de manera óptima. La perfusión intracardiaca ha sido descrita como una técnica que ofrece resultados superiores a los demás métodos de fijación de tejidos, ya que permite no solo una estabilidad adecuada de la muestra, sino también una limpieza profunda y un endurecimiento de los tejidos que permiten su manipulación posterior. A través de una variación de la técnica, es posible ocluir el principal suministro arterial de la región abdominal para mantener la perfusión directa del fijador en la región de interés, siendo esta la región maxilofacial y torácica.

## Abstract (en)

*In vivo* studies in dentistry require a high level of precision, since they involve the experimentation with a living organism, and further comprehensive histological analysis to validate the initial hypothesis. However, the process to obtained the histological slides to be studied is often wrongly minimized. In order to obtain high quality histological sections, which may be able to react favorably to more complex immunological techniques, it is necessary to preserve or "fix" the tissues of interest in an optimal manner. Intracardiac perfusion fixation has been described as a technique that offers superior results to other tissue fixation methods, allowing not only adequate sample stability, but also a deep cleansing and hardening of the tissues to allow further manipulation. Through a variation of the technique, it is possible to occlude the main arterial supply of the abdominal region to maintain direct perfusion of the fixator in the region of interest, such as the maxillofacial and thoracic region.

## INTRODUCCIÓN

Los estudios *in vivo* en odontología, ya sea empleando animales mayores o menores, exigen un alto nivel de responsabilidad y precisión, no solo en la etapa experimental, sino en las fases pre y posoperatorias. La experimentación con un ser viviente debe siempre tomar en cuenta las regulaciones y criterios éticos vigentes a nivel local e internacional, a fin de asegurar las mejores condiciones para una toma de muestras responsable que nos permita análisis histológico comprensivo y así validar la hipótesis original del estudio ^1^. 

Debido a la gran complejidad y el alto costo que implica este tipo de diseños experimentales, los investigadores, muchas veces, minimizan la etapa posoperatoria, es decir, se omite erróneamente la importancia de los procedimientos por los cuales se obtienen las láminas histológicas que serán analizadas. El largo proceso desde la obtención de las muestras hasta su tinción final implica una serie de protocolos en cada etapa que aseguren una adecuada imagen para el análisis histomorfométrico. Aunque cada paso es sumamente importante, estudiar la estructura y composición de los tejidos de la manera más confiable requiere la preservación óptima de estos ^2^^,^^3^. Tal preservación se da principalmente a través de la denominada “fijación” de tejidos.

El objetivo final de la fijación es conservar de manera uniforme los tejidos y las células en un estado idéntico al natural ^4^^,^^5^. De esta manera, se detiene toda alteración *post mortem*, para obtener un estado tisular que se asemeja a cuando el animal se encontraba vivo ^6^. Esto resulta de vital importancia debido a que las principales proteínas de las células que deseen ser visualizadas y estudiadas podrán reaccionar ante los procedimientos de inmunohistoquímica e inmunocitoquímica. Por ende, la fijación es uno de los pasos más importantes del proceso histológico. Una fijación de tejidos inadecuada puede resultar en células necróticas, presencia de células sanguíneas que interfieran en la visualización de otras estructuras, entre otros problemas.

Existe más de un método de fijación y una variedad de soluciones que pueden ser empleadas. Si bien es cierto que la literatura expresa que el método de fijación puede variar según el órgano objetivo ^3^, los resultados obtenidos a través de la fijación por perfusión intracardiaca sugieren que puede ser aplicable de manera completa o limitada a un área del cuerpo, y obtener resultados superiores que en los demás métodos de fijación ^7^^,^^8^.

## Fijación intracardiaca

La denominada fijación por perfusión intracardiaca es un método de fijación química que permite la administración efectiva y rápida de la solución fijadora a los tejidos, lo que a su vez permite una conservación óptima de estructuras celulares, proteínas, ácido desoxirribonucleico (ARN), etc. ^6^^,^^7^. Además, otorga estabilidad al tejido antes y después de la disección ^7^, pues lo endurece y, de este modo, mejora su posterior manipulación ^6^. La principal ventaja consiste en la perfusión directa del fijador a través del sistema circulatorio, por lo que puede alcanzar cada tejido del cuerpo utilizando la red vascular natural ^3^^,^^4^. En este método, el líquido fijador se introduce en el corazón, concretamente en el ventrículo izquierdo, y es distribuido a través del sistema arterial a los órganos diana ^2^. El conocimiento de la fisiología del sistema circulatorio del animal de experimentación, así como de su anatomía, es muy importante para que el investigador lleve a cabo esta técnica de manera exitosa. 

Si bien es cierto que otro método, denominado fijación por inmersión, es bastante utilizado debido a su practicidad, rapidez y a que no requiere un proceso de adiestramiento tan estricto como el de la perfusión intracardiaca, sus resultados pueden verse afectados por muchos factores, como el tamaño de la muestra, el tipo de tejido, la concentración de la solución fijadora, etc. Por ello, existe la posibilidad de que el líquido fijador no llegue a las capas celulares internas o que lo haga, pero no de manera uniforme ^2^^,^^8^.

La fijación por inmersión puede ser utilizada en casos en los que se trabaje con una buena cantidad de tejidos de menor tamaño y separados entre sí. La poca penetración de esta técnica no asegura una manipulación posterior sencilla de la muestra ^2^. El tiempo empleado también es un factor por considerar. Aun cuando es cierto que la fijación por inmersión requiere la disección previa de las muestras y su inmersión en el fijador, el tiempo en el que deben permanecer en este varía de 24 a 48 horas según el tamaño y cantidad de las muestras ^2^. La fijación por perfusión intracardiaca, por su parte, requiere el tiempo en el que 50 ml de fijador (cantidad aproximada para un roedor de 3 semanas) fluyen a través de la red vascular del animal y la inmersión posfijación no debe ser mayor a 24 horas ^9^^,^^10^.

Otra ventaja que posee la fijación intracardiaca es la limpieza adecuada de la sangre, tanto en los tejidos como en el sistema cardiovascular. El fijador removerá todo rastro de células sanguíneas y, de esta manera, mejorará la visualización de los resultados ^2^. Para utilizar la red vascular efectivamente, es importante considerar la presión de ingreso de la solución fijadora, igualándola con la presión fisiológica; de esta manera, evitamos lesionar la pared interna de los capilares ^4^^,^^11^.

Con frecuencia, la técnica de fijación por perfusión intracardiaca es aplicada con mayor frecuencia al cuerpo entero del animal de experimentación, aunque esto puede variar según el órgano objetivo. En el caso de los estudios *in vivo* en odontología, al concentrarnos en la región craniofacial, nos importa que la mayor cantidad del líquido fijador se concentre en la región superior del cuerpo. Por ende, surgió una modificación de la técnica convencional en la que se interrumpe la perfusión hacia el abdomen, mediante la oclusión de la arteria aorta descendente, que es el principal suministro sanguíneo a las estructuras por debajo del diafragma. Así, todo el líquido fijador se dirige de forma continua hacia la cabeza, la región torácica y las extremidades superiores ^3^.

El agente químico más utilizado para la perfusión de tejidos es el paraformaldehído (PFA), que es una forma estable, sólida y polimerizada del formaldehído ^6^^,^^8^^,^^12^. A partir de este compuesto, se prepara una solución que puede variar en su concentración (del 4 al 10%), sin afectar las pruebas histológicas posteriores, tal como el análisis inmunohistoquímico de las muestras. En comparación con la formalina tamponada neutra al 10%, las soluciones de paraformaldehído recién preparadas no contienen contaminantes (ácido fórmico y metanol). Los tejidos fijados con solución de PFA son bastante estables y pueden trabajarse posteriormente en parafina o criogenización. Las proteínas quedan inmovilizadas en los tejidos, lo cual permite un análisis preciso de la arquitectura celular ^4^. Esto último resulta de particular importancia para el análisis de tejidos complejos, como la pulpa dental, donde la detección de los odontoblastos es crítica para evaluar el estado de la pieza dental en modelos animales de injurias dentales severas ^13^.

## Protocolo de fijación intracardiaca

El protocolo abordado consiste en la fijación a través del sistema cardiovascular con la oclusión completa de la arteria aorta descendente y la vena cava inferior, para asegurar un flujo total y homogéneo en la región superior. La solución de PFA al 4% es almacenada a -20 °C, por lo que debe ser descongelada sumergiéndola en agua tibia una hora antes, hasta asegurar que esté completamente disuelta. Es recomendable que el agua utilizada no esté muy caliente, ya que los cambios abruptos de temperatura pueden romper el envase que contiene la solución de PFA o alterar su composición. En el caso de la fijación en ratones, son suficientes 50 ml de solución fijadora por cada animal para asegurar una perfusión óptima. Adicionalmente, 50 ml deben ser reservados para la inmersión posfijación, donde se guardarán las muestras por un plazo no mayor a 24 horas. El protocolo de la técnica de fijación por perfusión intracardiaca que se describe a continuación ha sido previamente reportado en múltiples publicaciones empleando modelos murinos (rata y ratón), principalmente de los tejidos dentales ^14^^-^^20^.

El primer paso para llevar a cabo la técnica de perfusión intracardiaca es anestesiar profundamente al animal de experimentación. Para esto, la solución anestésica se aplica de forma intraperitoneal, en el caso de rata y ratón, y se verifica que el animal haya alcanzado un plano profundo de sedación mediante el reflejo plantar, para dar inicio al acto operatorio ^1^. Es importante pesar cada animal previamente para la correcta dosificación de líquido anestésico.

**Figura 1:**
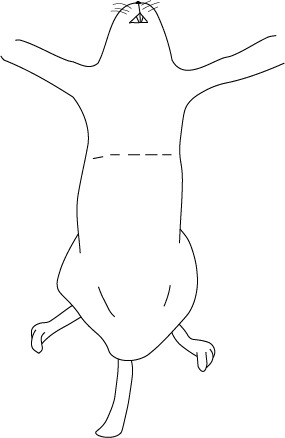
Incisión trasversal en piel

Se coloca al ratón en posición supina sobre una base firme y se asegura la inmovilidad las extremidades superiores. El procedimiento consiste en un ingreso hacia la cavidad torácica lo suficientemente amplio para visualizar el corazón, los pulmones y los principales vasos sanguíneos. La correcta remoción de la piel y, por consiguiente, del pelaje, es muy importante para que el procedimiento sea llevado de manera limpia y rápida (Figuras 1 y 2).

**Figura 2:**
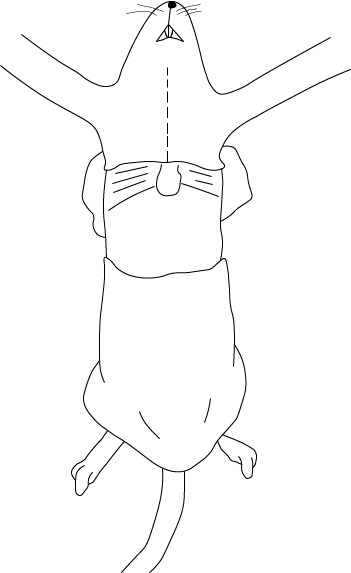
Incisión vertical en piel, a partir del borde de la incisión trasversal

**Figura 3:**
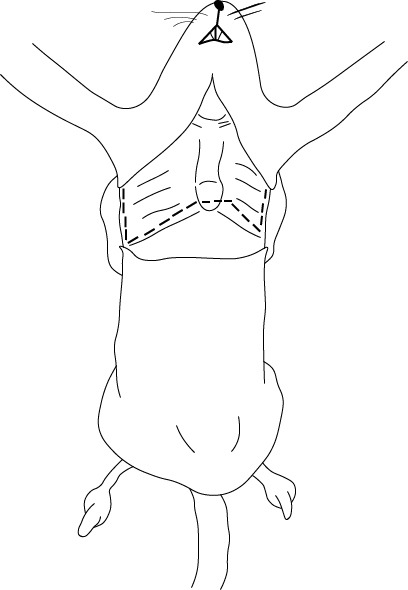
Corte trasversal en apófisis xifoides siguiendo el contorno de las costillas

El acceso a la cavidad torácica se realiza a través de incisiones en la piel con una tijera punta recta. Luego, con una tijera de osteotomía, se realiza un corte trasversal en la apófisis xifoides y se prolonga hacia los lados siguiendo el contorno de las costillas. Es importante utilizar una pinza para levantar el esternón y evitar daños en el corazón o los pulmones (Figura 3). Un acceso óptimo es el que se realiza con la menor cantidad de cortes posibles para dañar lo menos posible los tejidos y evitar la presencia de sangre que pueda dificultar la localización de los vasos sanguíneos.

**Figura 4:**
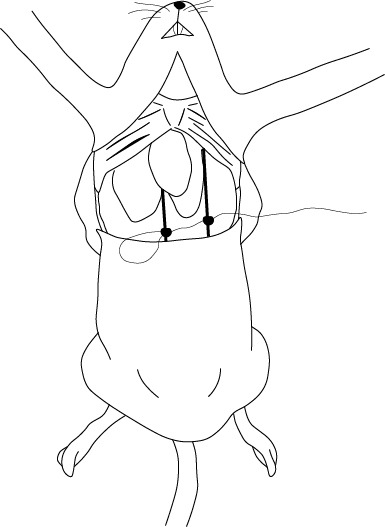
Oclusión completa de la arteria aorta descendente y de la vena cava inferior

El paso siguiente consiste en la oclusión arterial y venosa. Con la ayuda de una pinza, se debe retraer delicadamente el pulmón izquierdo hacia el centro para visualizar la arteria aorta descendente. Se utiliza seda blanca de sutura 2-0 para realizar un doble nudo simple y, con la misma seda, se realiza un doble nudo en la vena cava inferior. La pinza debe ser insertada por detrás de la arteria para que la seda se pueda deslizar. Una manipulación excesiva o una incorrecta inserción de la pinza puede resultar en una ruptura parcial o total de la arteria, lo que complica el procedimiento y, por consiguiente, el resultado final (Figura 4). 

**Figura 5:**
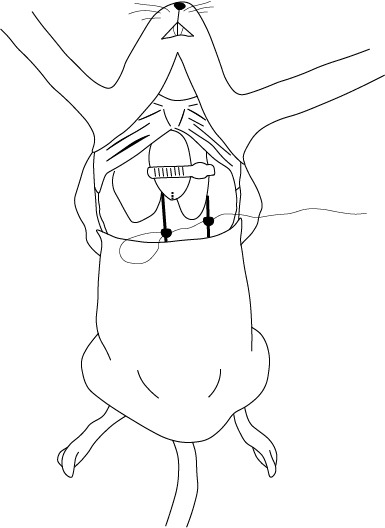
Incisión en ventrículo izquierdo del corazón, mientras es sujetado por una pinza arterial.

El paso final consiste en la perfusión del fijador a través del corazón. Este último debe ser sujetado con una pinza arterial y posicionado con el vértice apuntando hacia el diafragma. Se realiza una incisión con una tijera oftálmica en el ventrículo izquierdo y se introduce una cánula a través de la cual se suministrará, primero, solución salina, para remover la sangre de los vasos sanguíneos, y a continuación la solución de PFA al 4% (Figuras 5 y 6). Al mismo tiempo que se inicia la perfusión intracardiaca a través del ventrículo izquierdo, se realiza otra incisión, esta vez en la aurícula derecha, y se cortan las venas yugulares externas en la región cervical con una hoja de bisturí #11, para cerciorarnos del correcto fluido fuera del cuerpo.

**Figura 6:**
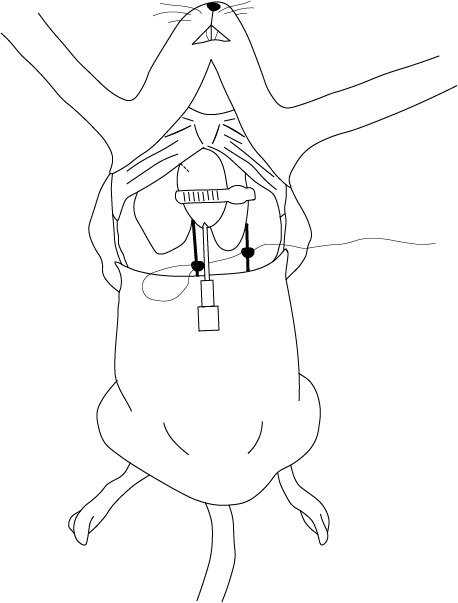
Escenario final de la fijación intracardiaca. Una cánula ingresa por el ventrículo izquierdo y se realiza una incisión en la aurícula derecha.

El color de los tejidos puede servir de guía. Si los pulmones palidecen o la solución empieza a fluir a través de nariz o boca, se debe modificar la posición de la cánula dentro del corazón, ya que esto es señal de que el líquido fijador está dirigiéndose a las vías aéreas. 

Al aumentar el flujo del fijador y liberar las extremidades, estas últimas deben levantarse inducidas por el flujo interno de solución de PFA al 4%. El movimiento de las extremidades superiores debe apuntar hacia delante del animal hasta quedar paralelas entre sí, para luego iniciar su endurecimiento. 

Tras haber completado la administración de 50 ml de solución fijadora en la porción superior del cuerpo del animal, se retira el instrumental de la caja torácica y se procede a la disección del maxilar con la ayuda de una tijera de osteotomía y una hoja de bisturí #25, a fin de cortar los músculos masetero y temporal. Para complementar el procedimiento, es recomendable sumergir el cráneo del animal de experimentación en aproximadamente 50 ml de solución de PFA al 4% por un mínimo de 12 horas adicionales, en condiciones de refrigeración (4 °C).

## CONCLUSIONES

La fijación intracardiaca ofrece múltiples ventajas sobre la fijación por inmersión en cuanto a la penetración del fijador, la limpieza de los tejidos y la conservación de la morfología, lo que se traduce finalmente en una mejoría en los resultados, que pueden ser visualizados microscópicamente a través de las tinciones y los ensayos inmunohistoquímicos.

## Fuente de financiamiento:

Autofinanciado
